# Targeted drug delivery of near IR fluorescent doxorubicin-conjugated poly(ethylene glycol) bisphosphonate nanoparticles for diagnosis and therapy of primary and metastatic bone cancer in a mouse model

**DOI:** 10.1186/s12951-016-0233-6

**Published:** 2016-12-05

**Authors:** S. Rudnick-Glick, E. Corem-Salkmon, I. Grinberg, S. Margel

**Affiliations:** Department of Chemistry, The Institute of Nanotechnology and Advanced Materials, Bar-Ilan University, 52900 Ramat Gan, Israel

**Keywords:** Bisphosphonates, Nanoparticles, NPs, Doxorubicin, Bone cancer, Targeted drug delivery

## Abstract

**Background:**

Most primary and metastatic bone tumors demonstrate increased osteoclast activity and bone resorption. Current treatment is based on a combination of surgery, radiotherapy and chemotherapy. Severe side effects are associated with chemotherapy due to use of high dosage and nonspecific uptake. Bisphosphonates have a strong affinity to Ca^2+^ ions and are widely used in the treatment of bone disorders.

**Results:**

We have engineered a unique biodegradable bisphosphonate nanoparticle (NPs) bearing two functional surface groups: (1) primary amine groups for covalent attachment of a dye/drug (e.g. NIR dye Cy 7 or doxorubicin); (2) bisphosphonate groups for targeting and chelation to bone hydroxyapatite. In addition, these engineered NPs contain high polyethyleneglycol (PEG) concentration in order to increase their blood half life time. In vitro experiments on Saos-2 human osteosarcoma cell line, demonstrated that at a tenth of the concentration, doxorubicin-conjugated bisphosphonate NPs achieved a similar uptake to free doxorubicin. In vivo targeting experiments using the NIR fluorescence bisphosphonate NPs on both Soas-2 human osteosarcoma xenograft mouse model and orthotopic bone metastases mCherry-labeled 4T1 breast cancer mouse model confirmed specific targeting. In addition, therapeutic in vivo experiments using doxorubicin-conjugated bisphosphonate NPs demonstrated a 40% greater inhibition of tumor growth in Saos-2 human osteosarcoma xenograft mouse model when compared to free doxorubicin.

**Conclusions:**

In this research we have shown the potential use of doxorubicin-conjugated BP NPs for the targeting and treatment of primary and metastatic bone tumors. The targeted delivery of doxorubicin to the tumor significantly increased the efficacy of the anti-cancer drug, thus enabling the effective use of a lower concentration of doxorubicin. Furthermore, the targeting ability of the BP NPs in an orthotopic xenograft mouse model reinforced our findings that these BP NPs have the potential to be used for the treatment of primary and metastatic bone cancer.

**Electronic supplementary material:**

The online version of this article (doi:10.1186/s12951-016-0233-6) contains supplementary material, which is available to authorized users.

## Background

It is well known that certain tumors have a predilection to metastasize to specific organs, for example breast, prostate, and lung cancers frequently metastasize to bone [1–3]. Most primary and metastatic bone tumors demonstrate increased osteoclast activity and bone resorption [4–6] which may lead to pathological fractures, hypercalcemia and pain [3].

Current treatment for both primary and metastatic bone tumors includes a combination of surgery, radiotherapy and chemotherapy [7, 8]. Although chemotherapy has increased the survival rate, poor bone blood supply [9] and non-tissue specificity necessitate the administration of high dosages, which consequently lead to severe side effects [7].

Bisphosphonates (BPs) are widely used in the treatment of bone resorption disorders such as osteoporosis [10], Paget disease [11] and primary and metastatic bone tumors [12]. BP is a stable chemical analog of pyrophosphate, in which the oxygen in the P–O–P bonds is replaced with a carbon (P–C–P) causing it to be enzymatically stable [11]. BP, like pyrophosphate, has a high affinity to the bone mineral hydroxyapatite, by generating either a bidentate or a tridentate chelation with the Ca^2+^ ion in the mineral [11, 13]. The BP chelation to bone is reversed in an acidic environment causing osteoclasts to internalize BP into membrane-bound vesicles during resorption causing a disruption in osteoclast activity [14, 15].

Over recent decades much research has been directed towards the development of nanoparticles (NPs) in the field of targeted drug delivery. Biodegradable NPs have great potential due to their sub-micron size, biocompatibility and enhanced permeability and retention effect [16, 17]. NPs provide protection from premature degradation and interaction with the biological environment, and enhance absorption and intracellular penetration of the drug to targeted tissue. In addition they enable greater control of the pharmacokinetics and drug body distribution [18]. There are several ways to utilize the NPs as a drug delivery system: either the NP itself is composed of the drug and attached to a targeting agent or the NP is composed of the targeting agent, and the therapeutic agents are encapsulated or covalently attached to its surface. Covalent biodegradable linkage (e.g., ester or amide bonds) confers the ability to accurately control the concentration of the drug attached and a known dosage can therefore be delivered and released at the targeted site [16]. Another application for the use of NPs is in the field of photonics for diagnostic imaging [19, 20].

Several research groups have utilized near-infrared (NIR) fluorescent dyes attached to NPs for in vivo imaging [19, 21]. NIR fluorescence (700–900 nm) exhibits low auto-fluorescence and higher penetration, compared to UV and visible light, due to lower light scattering by the biological tissue at this wavelength [22, 23].

In this research we have synthesized a biodegradable polymeric NP composed of a novel BP monomer, MA-PEG-BP (methacrylate polyethylene glycol BP), to target primary and secondary bone cancer, a primary amine containing monomer APMA (3-Aminopropyl)mathacrylamide) for the covalent attachment of a drug/dye to the surface of the NP and a crosslinker monomer tetra ethylene glycol diacrylate (TTEGDA). The incorporation of the high concentration of PEG endows the BP NPs with a relatively long blood half-life (5 h). This has been shown in vivo in a young mouse model using the NIR fluorescent Cy7-conjugated BP NPs [24]. In addition, we have demonstrated the bone targeting ability of the BP NPs [24] and the high toxicity of doxorubicin-conjugated BP NPs at low concentrations against osteosarcoma cells [25].

In this study, we have successfully illustrated the targeting ability of the BP NPs towards bone tumors in two in vivo mouse models. The NPs showed high selectivity for both osteosarcoma and breast cancer bone metastases. The therapeutic activity of the doxorubicin-conjugated BP was initially established using cell cycle studies on Soas-2 cells which demonstrated a greater uptake of the conjugated doxorubicin compared to free doxorubicin. In vivo studies using a Saos-2 subcutaneous tumor in Hsd:Athymic Nude-Foxn1^nu^ mice confirmed the enhanced bone tumor toxicity of the doxorubicin-conjugated BP NPs compared to free doxorubicin at a similar concentration.

## Methods

### Materials

The following analytical-grade chemicals were purchased from commercial sources and used without further purification: polyethylene glycol methacrylate (MA-PEG, Mn 360), TTEGDA, polyethylene glycol methacrylate ether (MA-PEG-OCH_3_, Mn 300), potassium persulfate, O-[(N-succinimidyl) succinyl-aminoethyl-O’-methylpolyethylene glycol (PEG-NHS, Mw 750), polyvinylpyrrolidone (PVP, Mw 360 K), sodium hydroxide (1 N), hydrochloric acid (1 N), anhydrous dichloromethane, anhydrous N,N-dimethylformamide, chromium oxide, isopropanol, magnesium sulfate (97%), triethylamine (99%), methanesulfonyl chloride, sodium chloride, sodium azide (99.5%), Tris(trimethylsilyl)phosphite, glycine and O,O’-bis[2-(*N*-succinimidyl-succinylamino)ethyl]polyethylene glycol (NHS-PEG-NHS,MW 3000) from Sigma (Rehovot, Israel); *N*-(3-aminopropyl) methacrylamide hydrochloride, (APMA) from Polysciences, Inc. (Warrington, PA); Dialysis membrane (1000 K-16MM), bicarbonate buffer (BB, 0.1 M, pH 8.4), sodium carbonate and sodium bicarbonate from Bio-Lab Ltd. (Jerusalem, Israel); Cy 7-NHS ester from Lumiprobe Corporation (Florida, USA); doxorubicin hydrochloride from Wonda science (Massachusetts, USA); Dulbecco’s phosphate-buffered saline (PBS), Dulbecco’s Minimum Essential Medium (DMEM), fetal bovine serum, glutamine, penicillin/streptomycin from Biological Industries (Bet Haemek, Israel); human osteosarcoma cell line Saos-2 and human colon carcinoma cell line SW620 from the American Type Culture Collection (Manassus, VA); Matrigel from Sigma (Germany); water was purified by passing deionized water through an Elgastat Spectrum reverse osmosis system (Elga Ltd., High Wycombe, UK).

### Synthesis of the BP NPs

BP NPs were prepared similarly to that described in the literature [26]. Briefly, 45 mg MA-PEG-BP [27, 28], 5 mg APMA and 50 mg TTEGDA (5% w/v total monomer concentration) were added to a vial containing 8 mg of the initiator potassium persulfate (8% w/w) and 20 mg of the stabilizer polyvinylpyrrolidone 360 K (1% w/v) dissolved in 2 mL of bicarbonate buffer (0.1 M). For the polymerization, the vial containing the mixture was purged with N_2_ to exclude air and then shaken at 83 °C for 8 h. The obtained BP NPs were washed of excess reagents by extensive dialysis cycles (cut-off of 1000 k) with purified water.

### Synthesis of the NIR fluorescent BP NPs

NIR fluorescent BP NPs were synthesized similarly to that described in the literature [26]. In brief, NIR fluorescent BP NPs were prepared by a reaction of the primary amino groups on the BP NPs with Cy7-NHS ester. Cy7-NHS ester (2 mg) was dissolved in 0.5 mL of anhydrous DMSO. 250 µL of the Cy7-NHS ester solution was then added to 5 mL of the BP NPs dispersion in 0.1 M bicarbonate buffer (2 mg/mL), and the reaction was stirred overnight at rt. Blocking of residual amine groups was then accomplished by adding 5 mg of O-[(*N*-succinimidyl) succinyl-aminoethyl-O’-methylpolyethylene glycol. The reaction was then stirred for 30 min at rt. The obtained NIR fluorescent-conjugated BP nanoparticles were then washed of excess reagents by extensive dialysis in water.

NIR fluorescent control nanoparticles possessing OCH_3_ groups instead of the BP groups were prepared similarly, substituting the monomer MA-PEG-BP for MA-PEG-OCH_3_.

The fluorescence following the conjugation of Cy7 to both the BP and control NPs was verified by both UV and fluorescence and was found to be similar.

### Synthesis of the doxorubicin-conjugated BP NPs

Doxorubicin-conjugated BP NPs were synthesized similarly to that described in the literature [25]. Doxorubicin-conjugated BP NPs were prepared by an initial reaction of the primary amine group on the BP NPs with NHS-PEG-NHS followed by the addition of doxorubicin. Briefly, NHS-PEG-NHS (10 mg) was dissolved in double distilled water (1 mL). 500 µL of the NHS-PEG-NHS solution was then added to 5 mL of the BP NPs dispersion in 0.1 M bicarbonate buffer (2 mg/mL), and the reaction was stirred at rt. After 10 min, 1 mg doxorubicin, initially dissolved in double distilled water, was added to the dispersion and was stirred for an additional 1 h. Blocking of residual amine groups was then accomplished by adding 50 mg of glycine to the doxorubicin BP NPs aqueous dispersion. The reaction was then stirred for a further 30 min at rt. The obtained doxorubicin-conjugated BP NPs were then washed of excess reagents by extensive dialysis (cut-off of 1000 k) in water. The concentration of the conjugated doxorubicin was determined using fluorescent intensity (λex 470 nm; λem 590 nm).

### Cell cultures

Saos-2 osteosarcoma cell line cultures were grown in Dulbecco’s Minimum Essential Medium supplemented with 10% heat-inactivated fetal bovine serum, 1% glutamine and 1% penicillin/streptomycin. 4T1 murine mammary adenocarcinoma cell line culture was grown in RPMI 1640 medium supplemented with 10% heat-inactivated fetal bovine serum, 1% glutamine and 1% penicillin/streptomycin. Cell lines were screened to ensure they remained mycoplasma-free using a myco-plasma detection kit.

### mCherry-infected 4T1 murine mammary adenocarcinoma cell line

Modified human embryonic kidney cell line GP2-293 was co-transfected with pRetroQ-mCherry-N1 Vector using the complementary Retro-X™ Universal system (Clontech, USA) to generate mCherry containing viral particles. 48 h following transfection, the pRetroQ-mCherry-N1 retroviral particles containing supernatant were collected. 4T1 murine mammary adenocarcinoma cells (ATCC, USA) were infected with the retroviral particle media, and 48 h following the infection, mCherry positive cells were selected by Puromycin (2 µg/ml) resistance [29].

### In vitro cell cycle studies

Cell cycle progression and apoptosis were analyzed by flow cytometry. For cell cycle analysis, Saos-2 cells (3 × 10^5^) were treated with doxorubicin-conjugated BP NPs [500, 250, 125, 50, 25 and 10 ng(doxo)/ml], doxorubicin and BP NPs (100 µg/ml) for 4 h. After incubation, cells were trypsinized, counted, and washed with culture medium. Cells were stained with Hoechst 33,342 solution according to the manufacturer’s protocol [30] and suspended in PBS. The cell suspension was analyzed by flow cytometry BD FACSAriaTM III (BD Biosciences, San Jose, CA, USA) with 488 and 405 nm lasers. A minimum of 10,000 cells were analyzed for each histogram generated. Gate SSC/FSC was used to exclude fragments and aggregates from the cell count. For multicolor flow cytometry the cells were treated with (1) doxorubicin (analyzed using Cy5) and (2) Hoechst (DAPI cell cycle analysis). In both cases untreated cells were used as control. Results were analyzed using FlowJo software according to the Dean–Jett–Fox model [31].

### Animal experiments

All mice were weighed prior to and throughout the experiments (20–25 g). Experiments were conducted on a total of 100 8 week old Hsd:Athymic Nude-Foxn1^nu^ female mouse model (Harlan Laboratories, Inc. Israel) and a total of 12 8 week old Balb/c female mice. Weight and tumor size were recorded weekly.

#### NIR fluorescent BP NPs targeting Saos-2 subcutaneous tumor in Hsd:Athymic Nude-Foxn1^nu^ mice

In order to determine the bone tumor targeting ability of the NIR fluorescent BP NPs experiments with Hsd:Athymic Nude-Foxn1^nu^ female mouse model (Harlan Laboratories, Inc. Israel) were carried out. Human osteosarcoma Saos-2 cells (3 × 10^6^) were suspended in 100 µL matrigel mix (1:1) and injected subcutaneously into the nude mice (n = 8). After a solid tumor was formed, three weeks post-subcutaneous injection, 100 µL Cy 7-conjugated BP NPs (0.1 mg/ml) suspended in PBS was IV injected via the tail vein. The mice were sacrificed at different time intervals and the tumors treated with NIR fluorescent BP and control NPs were studied by the Maestro II in vivo imaging system, 2D planar fluorescence imaging of small animals (Cambridge Research & Instrumentation, Inc., Woburn, MA, USA). The experiment was carried out twice.

The experiment was repeated with a subcutaneous tumor of SW620 human colon carcinoma cell line.

#### Saos-2 subcutaneous tumor in Hsd:Athymic Nude-Foxn1^nu^ mice treated with doxorubicin-conjugated BP NPs

In order to verify the doxorubicin-conjugated BP NPs anti-cancer activity, experiments on a Hsd:Athymic Nude-Foxn1^nu^ female mouse model (Harlan Laboratories, Inc. Israel) were performed. The Dox-BP NPs were tested at two different concentrations: 1 and 2 mg/ml with 5 and 10 µg (doxo)/ml (equivalent to 0.02 and 0.04 mg/kg doxorubicin per injection), respectively. Control groups consisted of mice injected with free doxorubicin 10 µg/ml (0.04 mg/kg) or BP NPs 2 mg/ml.

Human osteosarcoma Saos-2 cells (3 × 10^6^) were suspended in 100 µL matrigel mix (1:1) and injected subcutaneously into 8 week old female nude mice. The mice were randomly divided into 4 groups (n = 8 repeated twice): 0.2 mg doxorubicin-conjugated BP NPs (1 µg doxorubicin), 0.1 mg doxorubicin-conjugated BP NPs (0.5 µg doxo), 1 µg doxorubicin and 0.2 mg BP–NPs. After one week, the mice were IV injected via the tail vein with 100 µL of solution twice a week for 4 weeks. On the 30th day, mice were sacrificed using CO_2_ and tumors were extracted and weighed. The experiment was carried out twice using freshly synthesized NPs.

#### BP NPs ability to target mCherry-labeled 4T1 breast cancer bone metastases in Balb/C mouse model

8 week old Balb/c female mice (Harlan Laboratories, Inc. Israel) were injected intra-tibia with 5 × 10^5^ mCherry-labeled 4T1 cells suspended in matrigel [32]. One week post injection a tumor was present, and the mice were divided into two groups (n = 12). One group was treated with 100 µl of 0.1 mg/ml and the other group treated Cy7-conjugated BP nanoparticles or Cy7-conjugated control nanoparticles via IV injection into the tail vein. Mice were scanned after 72 h using Maestro in vivo imaging system (Cy5 filter: λex 587 nm, λem 610 nm and Cy7 filter: λex 710–760 nm, λem > 750 nm; Cy5 exposure time 0.5 s and Cy7 exposure 3 s), and then sacrificed. A Cy5 filter was used to image the mCherry expressing tumor and a Cy7 filter for the NPs. Images were analyzed using ImageJ software. The experiment was carried out twice using freshly synthesized NPs.

## Results

### Synthesis of non-fluorescent, NIR fluorescent and doxorubicin-conjugated BP NPs

**Fig. 1:**
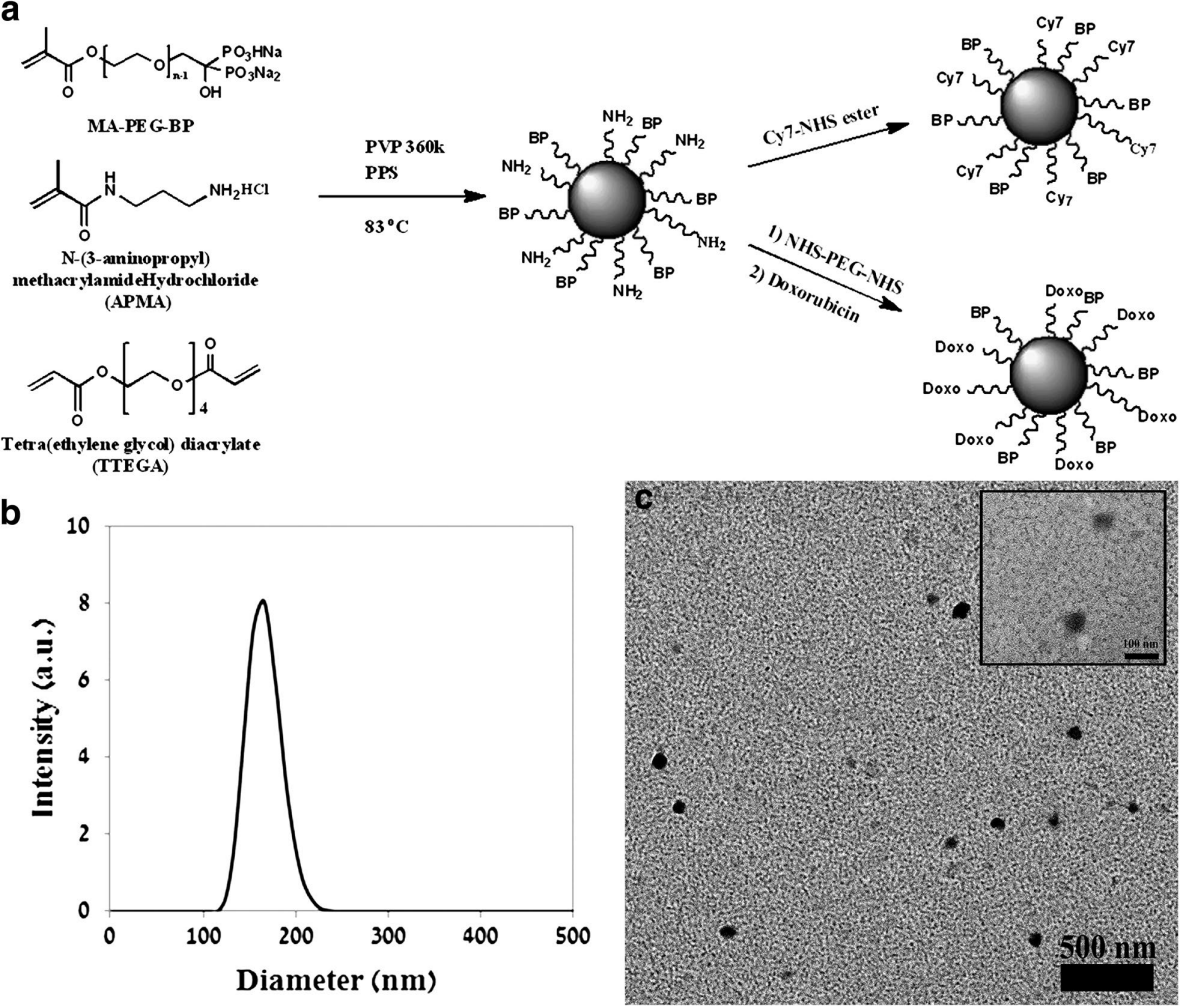
Synthesis scheme of BP NPs and conjugation of either Cy 7 or doxorubicin (**a**). Size histogram (**b**) and TEM image (**c**) of BP NPs

Functional crosslinked BP NPs of a dry diameter of 43 ± 5 nm and a hydrodynamic diameter of 160 ± 13 nm were prepared as described in the experimental part, by heterogeneous dispersion co-polymerization of the new BP monomer MA-PEG-BP [27] with the monomer APMA (3-Aminopropyl)mathacrylamide) and the crosslinker monomer TTEGDA (Fig. 1) [27]. These NPs were characterized using Dynamic light scattering and TEM and found to conform to those described in the literature [33]. The APMA monomer contains a primary amine group which allows for the covalent binding of a dye/drug to the surface of the particles as shown in Fig. 1. For optical imaging of bone tumor targeting we attached the NIR dye Cy7 to the surface of these particles [25, 33]. For therapeutic purposes doxorubicin was bound to the surface of the BP NPs through a PEG spacer, as described in the literature, per 1 mg of BP NPs 5 µg doxorubicin was conjugated [25]. The synthesis of both the BP NPs and the conjugation of doxorubicin are incredibly reproducible to that reported in the literature [25, 33]. Using the equation: $V = \frac{g}{d} = n \cdot \frac{4}{3} \cdot \pi \cdot r^{3}$ [d = density (1 g/ml); g = mass (1 g); r = radius (cm)], we were able to calculate the number (n) of BP NPs per mg (0.5 × 10^12^ particles), enabling us to determine the concentration of doxorubicin per BP NP as 1 × 10^−14^µg doxorubicin/NP. These NPs, due to their high content of BP, when administered by IV to chicken embryo model have been shown to specifically target bone tumor [25, 27, 33].

### In vitro activity of doxorubicin-conjugated BP NPs

**Fig. 2:**
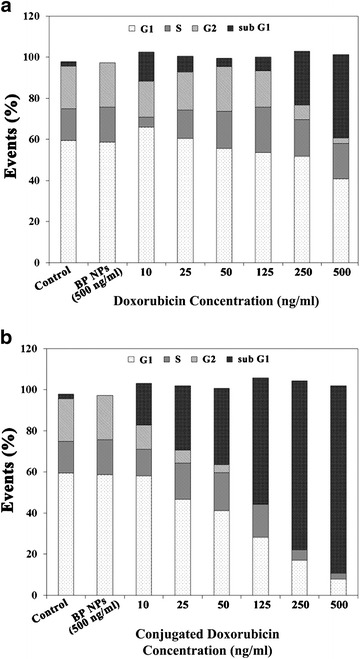
Cell cycle of Saos-2 cell treated with doxorubicin (**a**) and doxorubicin-conjugated BP NPs (**b**)

The effect of doxorubicin-conjugated BP NPs on cell cycle was compared to free doxorubicin and studied using flow cytometry. Human Saos-2 cells were incubated with free and conjugated doxorubicin at 10, 25, 50, 125, 250 and 500 ng/ml for 24 h. Free doxorubicin demonstrated no effect on cell cycle at the low dosages (10, 25 and 50 ng/ml). At 250 ng/ml 26% of cells were in sub G1 phase and at 500 ng/ml 40% were in sub G1 phase, Fig. 2a. However, treatment with doxorubicin-conjugated BP NPs exhibited a dose-dependent increase in sub G1 phase: 20% at 10 ng/ml, 31% at 25 ng/ml, 37% at 50 ng/ml, 61% at 125 ng/ml, 82% at 250 ng/ml and 91% at 500 ng/ml, Fig. 2b.

**Fig. 3:**
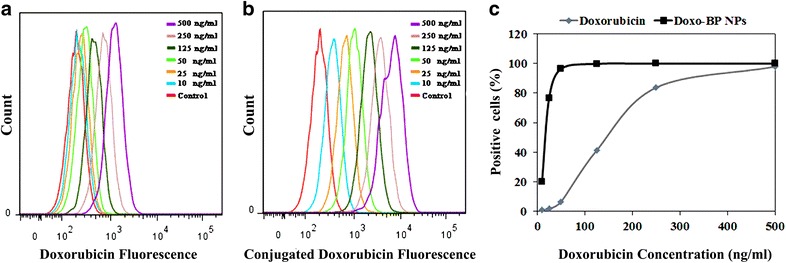
Intracellular fluorescence of Soas-2 cells treated with free doxorubicin (**a**) and doxorubicin-conjugated BP NPs (**b**) as a function of drug concentration. Graph comparing positive cell uptake of free and conjugated doxorubicin as a function of drug concentration(**c**)

Using flow cytometry, cell uptake of free and conjugated doxorubicin was studied. Figure 3 exhibits the intracellular fluorescence of free doxorubicin (Fig. 3a) and conjugated doxorubicin (Fig. 3b) as a function of drug concentration. Cells treated with doxorubicin-conjugated BP NPs exhibited a greater progressive shift in the fluorescence as a function of concentration. Figure 3c demonstrates the percentage of cells showing positive fluorescence due to doxorubicin as a function of concentration. At concentrations of 10, 25, 50, 125, 250 and 500 ng/ml the measured fluorescent uptake of free doxorubicin was 1.2, 1.7, 6.5, 41.2, 83.7 and 97.8%, respectively, whereas for doxorubicin-conjugated BP NPs the measured fluorescent uptake was 20, 76.7, 96.4, 99.8, 99.9 and 100%, respectively. Additional file 1: Figure S4 demonstrates that there is no change in the morphology of Saos-2 cells following 4 h treatment with doxorubicin-conjugated BP NPs (0.1 mg/ml).

### NIR fluorescent BP NPs targeting human osteosarcoma Saos-2 subcutaneous tumor in Hsd:Athymic Nude-Foxn1^nu^ mice

**Fig. 4:**
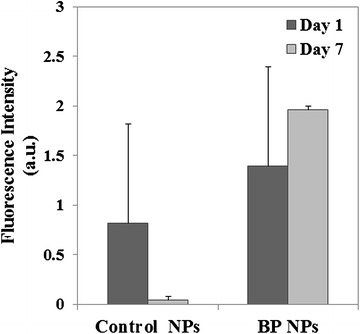
Targeting ability of Cy7-cojugated BP NPs compared to control NPs. Histogram of the difference in fluorescence between day 1 and day 7 of each NP. The fluorescence of the BP NPs remains constant indicating that they are retained in the area of the tumor, whereas the fluorescence of the control NPs is reduced, indicating that they have been cleared from the tumor area (analyzed by ImageJ software). *Error bars* represent standard deviation

In order to evaluate the ability of the Cy7-conjugated BP NPs to target a bone tumor, human osteosarcoma Saos-2 cells were injected subcutaneously into nude mice to induce an osteosarcoma xenograft. After a solid tumor was formed, Cy7-conjugated BP NPs and control NPs (0.1 mg/ml) were injected IV via the tail vein as described in the experimental part. The mice were sacrificed and the tumors were extracted after 1 and 7 days post-injection and analyzed using the Maestro II in vivo imaging system. One day post injection, both NPs were clearly visible within the tumors, though the tumors treated with Cy 7-conjugated BP NPs exhibited a slightly higher fluorescence (Fig. 4). 7 days post-injection the fluorescence of tumors treated with cy7-conjugated BP NPs was the same, whereas the fluorescence of the tumors treated with the cy7-conjugated control NPs decreased by 95%. The experiment was repeated using a tumor xenograft formed from SW620 human colon epithelial adenocarcinoma cell line (data not shown). No preferential uptake of cy7-conjugated BP NPs in comparison to the control NPs was evident.

### Therapeutic activity of doxorubicin-conjugated BP NPs on human osteosarcoma Saos-2 subcutaneous tumor in a nude mouse model

**Fig. 5:**
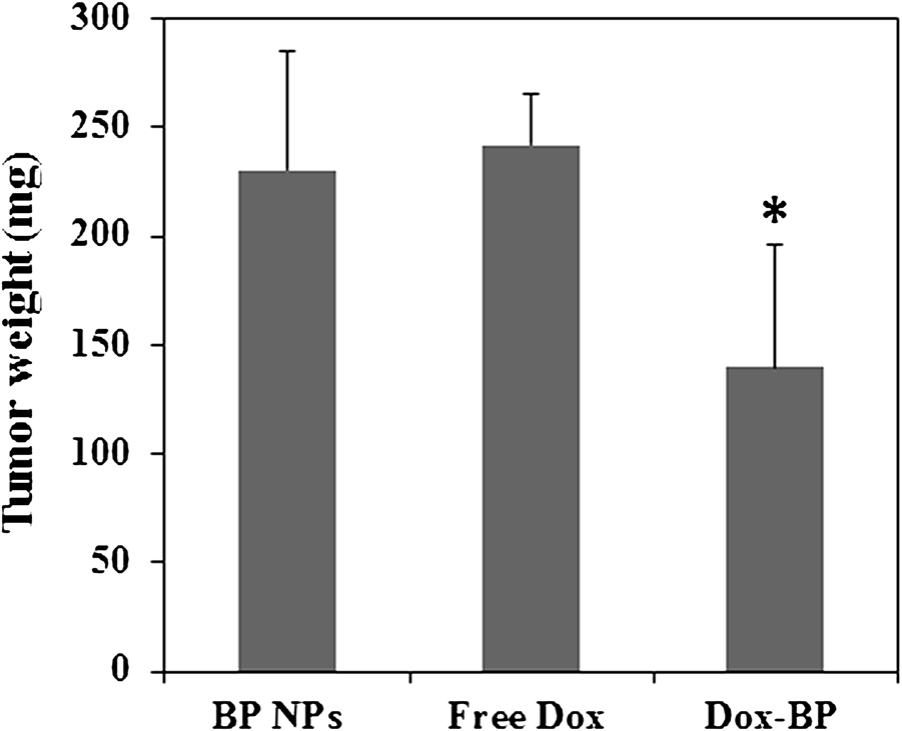
Effect on Saos-2 subcutaneous xenograft tumor in nude mouse model following bi-weekly IV injection for 30 days of doxorubicin-conjugated BP NPs (1 µg doxorubicin per injection), free doxorubicin (1 µg per injection) and non-conjugated BP NPs (0.2 mg per injection). *T test p < 0.05 and *error bars* represent standard deviation

Anti-cancer activity of doxorubicin-conjugated BP NPs in a Saos-2 subcutaneous xenograft tumor in a nude mouse model was studied (Fig. 5). Doxorubicin-conjugated BP NPs 1 µg doxorubicin per injection (equivalent 0.04 mg/kg doxorubicin per injection), free doxorubicin 1 µg per injection (0.04 mg/kg doxorubicin per injection) and non-conjugated BP NPs (0.2 mg per injection) were IV injected via the tail vein twice a week for 30 days and the effect on tumor growth was compared.

After mice were sacrificed the tumor was extracted and weighed. The average tumor weight for conjugated doxorubicin at 1 µg per injection was 140 mg, for free doxorubicin at 1 µg per injection was 242 mg, and for non-conjugated BP NPs at 0.2 mg per injection it was 230 mg, demonstrating a 40% difference between the free and conjugated doxorubicin (p < 0.05).

### NIR fluorescent BP NPs targeting breast cancer bone metastases in an orthotopic mCherry-labeled 4T1 tumor mouse model

**Fig. 6:**
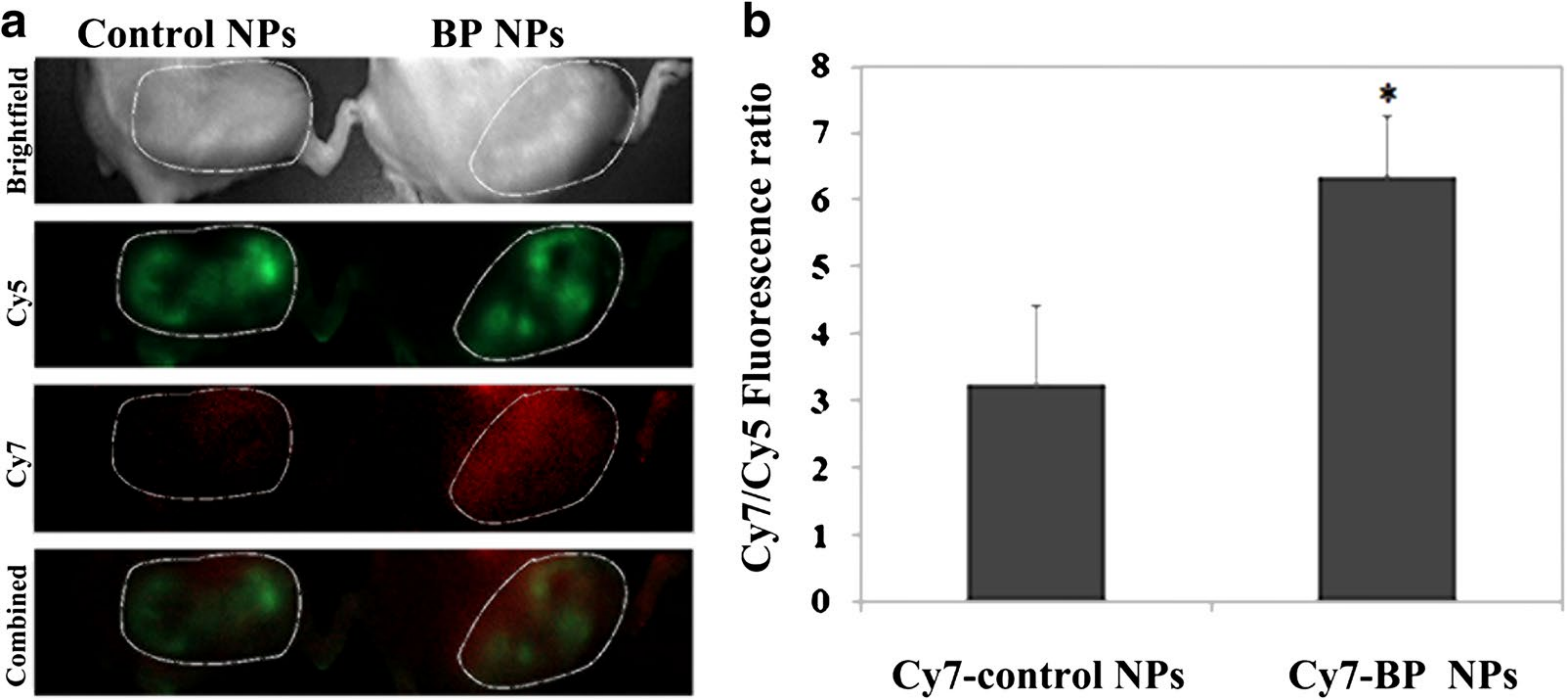
Balb/c female mice were injected intra-tibia with 4 × 10^5^ mCherry-labeled 4T1 cells. One week post injection the mice were treated with 0.1 mg/ml of either Cy7-conjugated BP nanoparticles or Cy7-conjugated control nanoparticles. Fluorescent images (**a**) and histogram of extracted tumors (**b**) after 72 h post injection of Cy7-conjugated BP nanoparticles. The *marked area* signifies the area of the tumor. Cy5 filter was used to image the mCherry expressing tumor along with Cy7 filter for the NPs

Balb/c female mice were injected intra-tibia with 5 × 10^5^ mCherry-labeled 4T1 cells [32]. One week post injection the mice were treated with 100 µl of 0.1 mg/ml of either Cy7-conjugated BP nanoparticles or Cy7-conjugated control nanoparticles via IV injection into the tail vein. Mice were scanned after 72 h using Maestro in vivo imaging system and images were analyzed using ImageJ software. The results obtained demonstrated that both NPs reach the tumor (Fig. 6). The Cy7-conjugated control NPs were present only at the periphery of the tumor. In contrast the Cy7-conjugated BP NPs were present throughout the whole tumor (Fig. 6a). Scans taken of the tumor, after the mice were sacrificed, showed that the fluorescence intensity of Cy7-conjugated BP NPs was significantly (p < 0.01) higher than the control group (Fig. 6b).

## Discussion

The results reported here demonstrate that the synthesis of the poly(ethylene glycol) bisphosphonate NPs is repeatable and produces NPs of the same size and the same concentration of bound doxorubicin as previously reported. It should be noted that crosslinking of the NPs during the coupling of doxorubicin to the NPs via the coupling reagent NHS-PEG-NHS was not observed, as shown in Additional file 1: Figure S1, probably due to the chosen experimental conditions (diluted aqueous dispersion and excess concentration of the NHS-PEG-NHS reagent as described in the experimental section). The high stability at physiological pH of the present NPs was confirmed by a negative zeta potential (−40 mV) as already demonstrated in our previous publications [25]. Furthermore, following the conjugation of doxorubicin to our novel poly(ethylene glycol) bisphosphonate NPs, a higher uptake and therapeutic effect was attained at lower concentrations than the free drug. Doxorubicin is known to affect the cell cycle [34, 35]. The cell cycle is divided into 3 main phases: G1 (growth and preparation for DNA division), S (duplication of DNA) and G2 (pre-mitotic). Apoptotic cells exhibit fractional DNA content known as sub G_1_ phase. The effect of doxorubicin-conjugated BP NPs on cell cycle was studied using flow cytometry to determine at what phase the treatment affects the cell. Human Saos-2 cells were incubated for 24 h with various concentrations of free and conjugated doxorubicin (10, 25, 50, 125, 250 and 500 ng/ml). When compared with the control group (the untreated cells), cell cycle analysis of both the cells treated with the free doxorubicin (Fig. 2a) and the doxorubicin-conjugated BP NPs (Fig. 2b) showed an increase in the number of unviable cells in the sub G_1_ phase, as described in the literature [34, 35]. However, when comparing these two groups, the doxorubicin-conjugated BP NPs group exhibited a greater number of cells in the sub G_1_ phase than the free doxorubicin group. At a low concentration of 10 ng/ml doxorubicin, the doxorubicin-conjugated BP NPs exhibited 20% cell death, whereas cells treated with free doxorubicin showed a comparable cell death (25%) only at a concentration that was 25 times greater, 250 ng/ml. In addition, at 500 ng/ml doxorubicin, 41% of the cells treated with free doxorubicin were in sub G1 phase, compared to 91% of cells treated with doxorubicin-conjugated BP NP. The increase in the number of unviable cells as a function of drug concentration could be explained by the greater intracellular concentration of doxorubicin, as indicated by the higher intracellular fluorescence of cells treated with doxorubicin-conjugated BP NPs (Fig. 3a) compared to the free doxorubicin (Fig. 3b), thus suggesting a dose-dependent cellular uptake. Figure 3c exhibits the number of cells which have internalized doxorubicin. It is evident that when doxorubicin is conjugated to BP NPs, maximum cellular uptake is achieved at a tenth of the concentration, 96% at 50 ng/ml, compared to the free doxorubicin 97% at 500 ng/ml. Since the uptake of doxorubicin-conjugated BP NPs was much greater than the free doxorubicin, at a low concentration, the toxic effect of the conjugated doxorubicin was greater and the internal doxorubicin fluorescence increases with the increase in concentration. The shift in the fluorescence (Fig. 3) can be explained by the increase in intracellular fluorescence which is directly related to the increase in intracellular concentration of doxorubicin. These results support previous experimental findings in which Saos-2 cells that had been treated for 48 h with 250 ng/ml doxorubicin exhibited 58% viability, whereas cells treated with doxorubicin-conjugated BP NPs exhibited 10% viability. A similar picture was also seen using U-2OS cell line, where 40 and 23% viability respectively was evident [25].

It has been reported in the literature that Saos-2 cells have osteoblastic properties and form a calcified matrix. In addition, it has been demonstrated that Saos-2 xenografts form an osteoid matrix, similar to woven bone, consistent with osteosarcoma [36–39]. The results obtained for the NIR fluorescent Cy 7-conjugated BP NPs targeting ability to Saos-2 subcutaneous tumor showed that 1 day post-injection (0.1 mg/ml), a higher fluorescence was seen compared to the control NPs (Fig. 4). Seven days post-injection revealed that the fluorescence of Cy 7-conjugated BP NPs remained constant within the tumor, whereas the fluorescence of the control NPs dramatically decreased by 95%. The tumor fluorescence could not be due to cleaved Cy 7, since Cy 7 is rapidly eliminated [40, 41]. The uptake of the Cy 7-conjugated BP NPs within the tumor and their retention over time can be explained by the high affinity of the BP functional group to Ca^2+^ ions, thereby endowing these BP NPs with the ability to preferentially target human osteosarcoma Saos-2 tumor. The initial uptake of the control NPs could be explained by the EPR effect, where increased vascular permeability leads to leakage of the blood-borne NPs into the tumor [42–45]. The specific uptake of BP NPs in osteosarcoma tumor was confirmed on repetition of the experiment, using a tumor xenograft formed from SW620 human colon epithelial adenocarcinoma cell line, where no preferential uptake was evident.

An earlier published study demonstrated the anti-cancer activity of the doxorubicin-conjugated BP NPs in vitro and in ovo, in a chicken embryo CAM tumor model [25]. In the current study we further investigated the anti-cancer activity of these doxorubicin-conjugated BP NPs in a Saos-2 subcutaneous xenograft tumor in a nude mouse model.

Results obtained demonstrate no change in tumor weight following treatment with the free doxorubicin 1 µg per injection (0.04 mg/kg), thereby indicating no toxic effect on the Saos-2 subcutaneous tumor growth. However, tumors treated with doxorubicin-conjugated BP NPs 0.5 µg doxorubicin per injection (0.02 mg/kg) exhibited a 40% reduction in weight after 30 days’ biweekly injection (Fig. 5). It has been reported in the literature that the therapeutic effect of a series of IV injected doxorubicin is achieved at 5 mg/kg [46]. In our study we were able to demonstrate a toxic effect of doxorubicin at a concentration 100 times lower. The enhanced toxic effect of the doxorubicin when conjugated to BP NPs can only be explained by a higher concentration of doxorubicin delivered to the tumor by the specific targeting of the BP NPs. Hence, these results support the findings of the previous experiment and provide further evidence that the BP NPs specifically target osteosarcoma tumors, indicating the potential of doxorubicin-conjugated BP NPs for use as a drug delivery system in the treatment of osteosarcoma.

One major disadvantage of subcutaneous xenograft tumor models is that the microenvironment of the implanted tumor does not reproduce the environment in which the tumor grows [47–49]. However, it has been reported that a variety of tumor tissues when administered into the appropriate anatomical site in a mouse model, will often metastasize to similar locations as does the tumor in humans [32, 49, 50]. Therefore, after establishing the potential of the BP NPs targeting and therapeutic ability in a subcutaneous xenograft mouse model we continued our study in an orthotopic model. Breast cancer commonly metastasizes to bone [51–53] and we therefore orthotopically implanted mCherry-labeled 4T1 mammary carcinoma cells intra-tibially in Balb/c mice [32] in order to produce bone tumor. Hence, this model provided an appropriate microenvironment for the evaluation of the tumor targeting ability of the BP NPs in bone.

Results obtained, following IV injection of Cy7-conjugated BP NPs and Cy7-conjugated control NPs (at the same initial fluorescence intensity), clearly indicated that both NPs reached the tumor area. The control NPs were seen to exhibit passive targeting which can be attributed to the EPR effect [43]. In contrast, the BP NPs demonstrated active tumor targeting as a result of the bone resorption, caused by the tumor invasion into the bone. Thus, we have shown that these BP NPs directly target bone tumors and have the potential to be utilized in the diagnosis and treatment of both primary and metastatic bone cancers.

Previous studies have shown that the combination of doxorubicin and nitrogen containing BPs lead to an improved therapeutic effect [54–56]. In this study however, the BPs in the engineered BP NPs did not contain nitrogen and showed no toxic effect on bone tumors and therefore were used only as a bone tumor targeting agent. We have not determined if a synergistic effect exists between the BP NPs combined with doxorubicin and further research should be carried out.

There are several postulated mechanisms by which doxorubicin might cause cellular damage. At present the two most accepted theories are either intercalation of the doxorubicin into DNA, which leads to disruption of topoisomerase-II-mediated DNA repair and/or damage to cellular membranes by generation of free radicals [57]. However, the mechanism by which the surface bound doxorubicin NPs acts on cancer cells has not yet been determined. It is unclear whether the doxorubicin-conjugated NPs directly cause cellular damage or whether the cleavage of doxorubicin from the NPs is essential for cytotoxicity.

To summarize, we have shown the potential use of doxorubicin-conjugated BP NPs for the targeting and treatment of osteosarcoma. These doxorubicin-conjugated BP NPs, due to their high affinity to Ca^2+^ ions, enable the delivery of doxorubicin directly to the tumor. We have confirmed that conjugation of doxorubicin to BP NPs significantly increases the anti-cancer activity of the drug against Soas-2 osteosarcoma cells, by increasing the uptake of the anti-cancer drug in the cell compared to the free drug. This was further investigated on human osteosarcoma Saos-2 subcutaneous tumor in a nude mouse model, where we verified the affinity of the doxorubicin-conjugated BP NPs to osteosarcoma tumors and their therapeutic activity on them. The results obtained have revealed that the targeted delivery of doxorubicin significantly increased the efficacy of the anti-cancer drug, thus enabling the effective use of a lower concentration of doxorubicin. The targeting ability of the BP NPs in an orthotopic xenograft mouse model reinforced our findings that these BP NPs have the potential to be used for the treatment of primary and metastatic bone cancer.

The intention of this study was to assess the short term targeting and therapeutic potential of these novel BP NPs. We expect that since the NPs are highly hydrophilic and contain esteric bonds, the NPs are biodegradable. Our future plans include evaluating their biodegradability. In addition, we intend to extend our investigation of the therapeutic activity of the doxorubicin-conjugated BP NPs in an in vivo osteosarcoma orthotopic model and in a genetically engineered mouse (GEM) model.

## Additional file

**Additional file 1.** Additional figures.

## Abbreviations

BPs: bisphosphonates
NPs: nanoparticles
MA-PEG: polyethylene glycol methacrylate
TTEGDA: tetraethylene glycol diacrylate
Doxo: doxorubicin
NHS-PEG-NHS: O,O′-bis[2-(*N*-succinimidyl-succinylamino)ethyl]polyethylene glycol
APMA: *N*-(3-aminopropyl) methacrylamide hydrochloride
PBS: Dulbecco’s phosphate-buffered saline
NIR: near IR
TEM: transmission electron microscopy
PEG: polyethylene glycol

## References

[1] Buchbender C, Heusner TA, Lauenstein TC, Bockisch A, Antoch G (2012). Oncologic PET/MRI, part 2: bone tumors, soft-tissue tumors, melanoma, and lymphoma. J Nucl Med.

[2] Zhang XHF, Jin X, Malladi S, Zou Y, Wen YH, Brogi E, Smid M, Foekens JA, Massagué J (2013). Selection of bone metastasis seeds by mesenchymal signals in the primary tumor stroma. Cell.

[3] Ell B, Mercatali L, Ibrahim T, Campbell N, Schwarzenbach H, Pantel K, Amadori D, Kang Y (2013). Tumor-induced osteoclast miRNA changes as regulators and biomarkers of osteolytic bone metastasis. Cancer Cell.

[4] Scott SJ, Prakash P, Salgaonkar V, Jones PD, Cam RN, Han M, Rieke V, Burdette EC, Diederich CJ, Ryan TP (2013). Interstitial ultrasound ablation of tumors within or adjacent to bone contributions of preferential heating at the bone surface. SPIE BiOS.

[5] Coleman RE (2001). Metastatic bone disease: clinical features, pathophysiology and treatment strategies. Cancer Treat Rev.

[6] Coleman R (2016). Treatment of metastatic bone disease and the emerging role of radium-223. Semin Nucl Med.

[7] Longhi A, Errani C, De Paolis M, Mercuri M, Bacci G (2006). Primary bone osteosarcoma in the pediatric age: state of the art. Cancer Treat Rev.

[8] Coleman RE, McCloskey EV (2011). Bisphosphonates in oncology. Bone.

[9] Roche B, Vanden-Bossche A, Normand M, Malaval L, Vico L, Lafage-Proust M-H (2013). Validated Laser Doppler protocol for measurement of mouse bone blood perfusion-response to age or ovariectomy differs with genetic background. Bone.

[10] Whitaker M, Guo J, Kehoe T, Benson G (2012). Bisphosphonates for osteoporosis—where do we go from here?. N Engl J Med.

[11] Russell RGG (2011). Bisphosphonates: the first 40 years. Bone.

[12] Spina A, Sorvillo L, Di Maiolo F, Esposito A, D’Auria R, Di Gesto D, Chiosi E, Naviglio S (2013). Inorganic phosphate enhances sensitivity of human osteosarcoma U2OS cells to doxorubicin via a p53-dependent pathway. J Cell Physiol.

[13] Nancollas GH, Tang R, Phipps RJ, Henneman Z, Gulde S, Wu W, Mangood A, Russell RGG, Ebetino FH (2006). Novel insights into actions of bisphosphonates on bone: differences in interactions with hydroxyapatite. Bone.

[14] Coxon FP, Thompson K, Roelofs AJ, Ebetino FH, Rogers MJ (2008). Visualizing mineral binding and uptake of bisphosphonate by osteoclasts and non-resorbing cells. Bone.

[15] De Rosa G, Misso G, Salzano G, Caraglia M (2013). Bisphosphonates and cancer: what opportunities from nanotechnology?. J Drug Deliv.

[16] Wilczewska AZ, Niemirowicz K, Markiewicz KH, Car H (2012). Nanoparticles as drug delivery systems. Pharmacol Rep.

[17] Chen W, Meng F, Cheng R, Deng C, Feijen J, Zhong Z (2014). Advanced drug and gene delivery systems based on functional biodegradable polycarbonates and copolymers. J Control Release.

[18] Peer D, Karp JM, Hong S, Farokhzad OC, Margalit R, Langer R (2007). Nanocarriers as an emerging platform for cancer therapy. Nat Nanotechnol.

[19] Yezhelyev MV, Gao X, Xing Y, Al-Hajj A, Nie S, O’Regan RM (2006). Emerging use of nanoparticles in diagnosis and treatment of breast cancer. Lancet Oncol.

[20] Cohen S, Pellach M, Kam Y, Grinberg I, Corem-Salkmon E, Rubinstein A, Margel S (2013). Synthesis and characterization of near IR fluorescent albumin nanoparticles for optical detection of colon cancer. Mater Sci Eng C Mater Biol Appl.

[21] Fortina P, Kricka LJ, Graves DJ, Park J, Hyslop T, Tam F, Halas N, Surrey S, Waldman SA (2007). Applications of nanoparticles to diagnostics and therapeutics in colorectal cancer. Trends Biotechnol.

[22] Askinadze N, Gluz E, Ziv O, Mizrahi DM, Margel S (2013). Engineering of new crosslinked functional PEG micrometer-sized particles of narrow size distribution for enzyme immobilization. Polymer (Guildf).

[23] Amiot CL, Xu S, Liang S, Pan L, Zhao JX (2008). Near-infrared fluorescent materials for sensing of biological targets. Sensors.

[24] Rudnick-Glick S, Corem-Salkmon E, Grinberg I, Yehuda R, Margel S (2015). Near IR fluorescent conjugated poly(ethylene glycol)bisphosphonate nanoparticles for in vivo bone targeting in a young mouse model. J Nanobiotechnol.

[25] Rudnick-Glick S, Corem-Salkmon E, Grinberg I, Gluz E, Margel S (1022). Doxorubicin-conjugated bisphosphonate nanoparticles for the therapy of osteosarcoma. JSM Nanotechnol Nanomed.

[26] Gluz E, Grinberg I, Corem Salkmon E, Mizrahi D, Margel S, Corem-Salkmon E, Mizrahi D, Margel S (2013). Engineering of new crosslinked near-infrared fluorescent polyethylene glycol bisphosphonate nanoparticles for bone targeting. J Polym Sci Part A Polym Chem.

[27] Gluz E, Mizrahi DM, Margel S (2013). Synthesis and characterization of new poly(ethylene glycol)bisphosphonate vinylic monomer and non-fluorescent and NIR-fluorescent bisphosphonate micrometer-sized particles. Polymer (Guildf).

[28] Mizrahi DM, Ziv-Polat O, Perlstein B, Gluz E, Margel S (2011). Synthesis, fluorescence and biodistribution of a bone-targeted near-infrared conjugate. Eur J Med Chem.

[29] Markovsky E, Baabur Cohen H, Eldar Boock A, Omer L, Tiram G, Ferber S, Ofek P, Polyak D, Scomparin A, Satchi Fainaro R (2012). Administration, distribution, metabolism and elimination of polymer therapeutics. J Control Release.

[30] Chazotte B (2011). Labeling nuclear DNA with hoechst 33342. Cold Spring Harb Protoc.

[31] Fox MH (1980). A model for the computer analysis of synchronous DNA distributions obtained by flow cytometry. Cytometry.

[32] Miller K, Eldar Boock A, Polyak D, Segal E, Benayoun L, Shaked Y, Satchi Fainaro R (2011). Antiangiogenic antitumor activity of HPMA copolymer-paclitaxel-alendronate conjugate on breast cancer bone metastasis mouse model. Mol Pharm.

[33] Gluz E, Rudnick-Glick S, Mizrahi DM, Chen R, Margel S (2014). New biodegradable bisphosphonate vinylic monomers and near infrared fluorescent nanoparticles for biomedical applications. Polym Adv Technol.

[34] Siu WY, Yam CH, Poon RY (1999). G1 versus G2 cell cycle arrest after adriamycin-induced damage in mouse Swiss3T3 cells. FEBS Lett.

[35] Lüpertz R, Wätjen W, Kahl R, Chovolou Y (2010). Dose- and time-dependent effects of doxorubicin on cytotoxicity, cell cycle and apoptotic cell death in human colon cancer cells. Toxicology.

[36] Rodan SB, Imai Y, Thiede MA, Wesolowski G, Thompson D, Bar Shavit Z, Shull S, Mann K, Rodan GA (1987). Characterization of a human osteosarcoma cell line (Saos-2) with osteoblastic properties. Cancer Res.

[37] Mills J, Matos T, Charytonowicz E, Hricik T, Castillo-Martin M, Remotti F, Lee FY, Matushansky I (2009). Characterization and comparison of the properties of sarcoma cell lines in vitro and in vivo. Hum Cell.

[38] McQuillan D, Richardson MD, Bateman JF (1995). Matrix deposition by a calcifying human osteogenic sarcoma cell line (Saos-2). Bone.

[39] Prideaux M, Wijenayaka AR, Kumarasinghe DD, Ormsby RT, Evdokiou A, Findlay DM, Atkins GJ (2014). Saos-2 Osteosarcoma cells as an in vitro model for studying the transition of human osteoblasts to osteocytes. Calcif Tissue Int.

[40] Zou P, Xu S, Povoski SP, Wang A, Johnson MA, Martin EW, Subramaniam V, Xu R, Sun D (2010). Near-infrared fluorescence labeled anti-TAG-72 monoclonal antibodies for tumor imaging in colorectal cancer xenograft mice. Mol Pharm.

[41] Botz B, Bölcskei K, Kemény Á, Sándor Z, Tékus V, Sétáló G, Csepregi J, Mócsai A, Pintér E, Kollár L, Helyes Z (2015). Hydrophobic cyanine dye-doped micelles for optical in vivo imaging of plasma leakage and vascular disruption. J Biomed Opt.

[42] Greish K (2010). Enhanced permeability and retention (EPR) effect for anticancer nanomedicine drug targeting. Methods Mol Biol.

[43] Lammers T, Kiessling F, Hennink WE, Storm G (2012). Drug targeting to tumors: principles, pitfalls and (pre-) clinical progress. J Control Release.

[44] Alexis F, Pridgen E, Molnar LK, Farokhzad OC (2008). Factors affecting the clearance and biodistribution of polymeric nanoparticles. Mol Pharm.

[45] Fullstone G, Wood J, Holcombe M, Battaglia G (2015). Modelling the transport of nanoparticles under blood flow using an agent-based approach. Sci Rep.

[46] Kubo T, Shimose S, Matsuo T, Sakai A, Ochi M (2008). Efficacy of a nitrogen-containing bisphosphonate, minodronate, in conjunction with a p38 mitogen activated protein kinase inhibitor or doxorubicin against malignant bone tumor cells. Cancer Chemother Pharmacol.

[47] Johnson JI, Decker S, Zaharevitz D, Rubinstein LV, Venditti JM, Schepartz S, Kalyandrug S, Christian M, Arbuck S, Hollingshead M, Sausville EA (2001). Relationships between drug activity in NCI preclinical in vitro and in vivo models and early clinical trials. Br J Cancer.

[48] Morton CL, Houghton PJ (2007). Establishment of human tumor xenografts in immunodeficient mice. Nat Protoc.

[49] Varna M, Bertheau P, Legrès LG (2014). Tumor microenvironment in human tumor xenografted mouse models. J Anal Oncol.

[50] Bibby MC (2004). Orthotopic models of cancer for preclinical drug evaluation: advantages and disadvantages. Eur J Cancer.

[51] Azim H, Azim HA (2013). Targeting RANKL in breast cancer: bone metastasis and beyond. Expert Rev Anticancer Ther.

[52] Petrut B, Trinkaus M, Simmons C, Clemons M (2008). A primer of bone metastases management in breast cancer patients. Curr Oncol.

[53] Oster G, Lamerato L, Glass AG, Richert-Boe KE, Lopez A, Chung K, Richhariya A, Dodge T, Wolff GG, Balakumaran A, Edelsberg J (2013). Natural history of skeletal-related events in patients with breast, lung, or prostate cancer and metastases to bone: a 15-year study in two large US health systems. Support Care Cancer.

[54] Camirand A, Fadhil I, Luco A-L, Ochietti B, Kremer RB (2013). Enhancement of taxol, doxorubicin and zoledronate anti-proliferation action on triple-negative breast cancer cells by a PTHrP blocking monoclonal antibody. Am J Cancer Res.

[55] Riganti C, Castella B, Kopecka J, Campia I, Coscia M, Pescarmona G, Bosia A, Ghigo D, Massaia M, Swanson K, Hohl R, Clendening J, Pandyra A, Boutrosa P, El Ghamrasni S, Khosravi F, Freed-Pastor W, Mizuno H, Zhao X, Langerød A, Moon SH, Gottesman M, Fojo T, Bates S, Troost J, Lindenmaie J, Haefeli W, Weiss J, Schmidmaier R, Baumann P (2013). Zoledronic acid restores doxorubicin chemosensitivity and immunogenic cell death in multidrug-resistant human cancer cells. PLoS ONE.

[56] Ottewell PD, Woodward JK, Lefley DV, Evans CA, Coleman RE, Holen I, Chirgwin J, Mohammad K, Guise T, Green J, Amin D, Cornell S, Gustafson S, Van Beek E, Pieterman E, Cohen L, Lowick C, Papapoulos S, Dunford J, Thompson K, Coxon F, Coxon F, Helfrich M, Hof RV, Rogers M, Gordon S, Benford H, Benford H, McGowan N, Helfrich M (2009). Anticancer mechanisms of doxorubicin and zoledronic acid in breast cancer tumor growth in bone. Mol Cancer Ther.

[57] Thorn CF, Oshiro C, Marsh S, Hernandez-Boussard T, McLeod H, Klein TE, Altman RB (2011). Doxorubicin pathways: pharmacodynamics and adverse effects. Pharmacogenet Genomics.

