# Supplementary material for: Targeted drug delivery of near IR fluorescent doxorubicin-conjugated poly(ethylene glycol) bisphosphonate nanoparticles for diagnosis and therapy of primary and metastatic bone cancer in a mouse model
Source: J Nanobiotechnology. 2016 Dec 5;14:80. doi: 10.1186/s12951-016-0233-6 (PMC5139040; doi:10.1186/s12951-016-0233-6)

**Targeted drug delivery of near IR fluorescent doxorubicin-conjugated poly(ethylene glycol) bisphosphonate nanoparticles for diagnosis and therapy of primary and metastatic bone cancer in a mouse model**

Rudnick-Glick S., Corem-Salkmon E., Grinberg I. and Margel S*

**The Institute of Nanotechnology and Advanced Materials, Department of Chemistry, Bar-Ilan University, Ramat-Gan 52900, Israel**

**Supporting information**

**Figure S1. DLS analysis of doxorubicin-conjugated BP NPs**

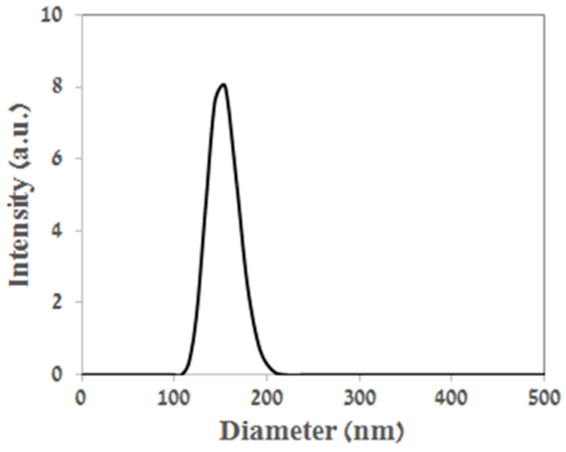

**Figure S2. Cell cycle analysis - Gating strategy.** Gate SSC/FSC was used to exclude from the cell count fragments and aggregates. For multicolor flow cytometry the cells were treated with A) doxorubicin (analyzed using Cy5) and B) Hoechst (DAPI cell cycle analysis). In both cases untreated cells were used as control. Results were analyzed using FlowJo software according to the Dean-Jett- Fox model.

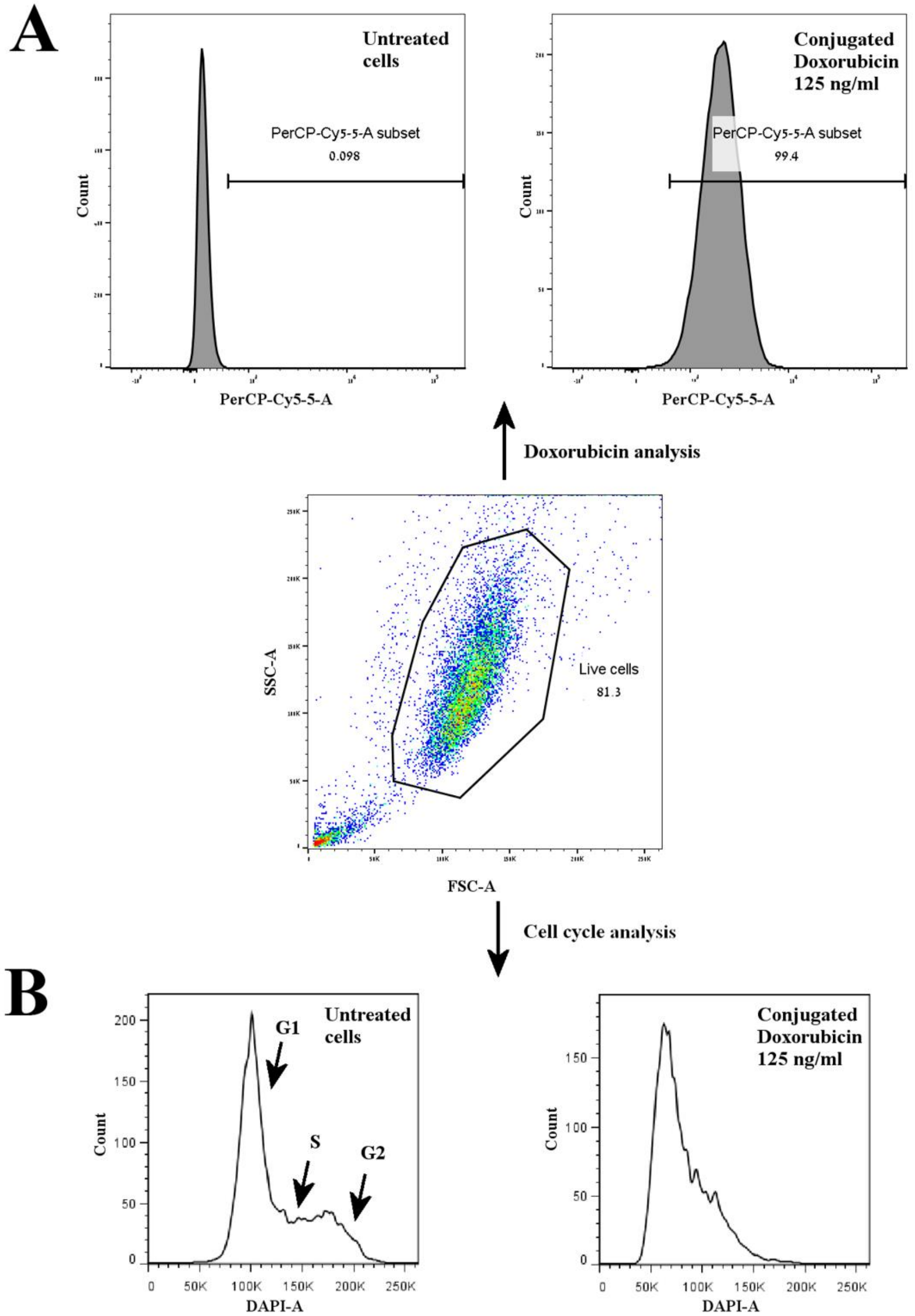

**Figure S3.** A) Fluorescent images of extracted tumors (delineated areas) after 1 and 7 days post IV injection with Cy7-cojugated BP NPs and control NPs. B) Measurements method using imageJ software.

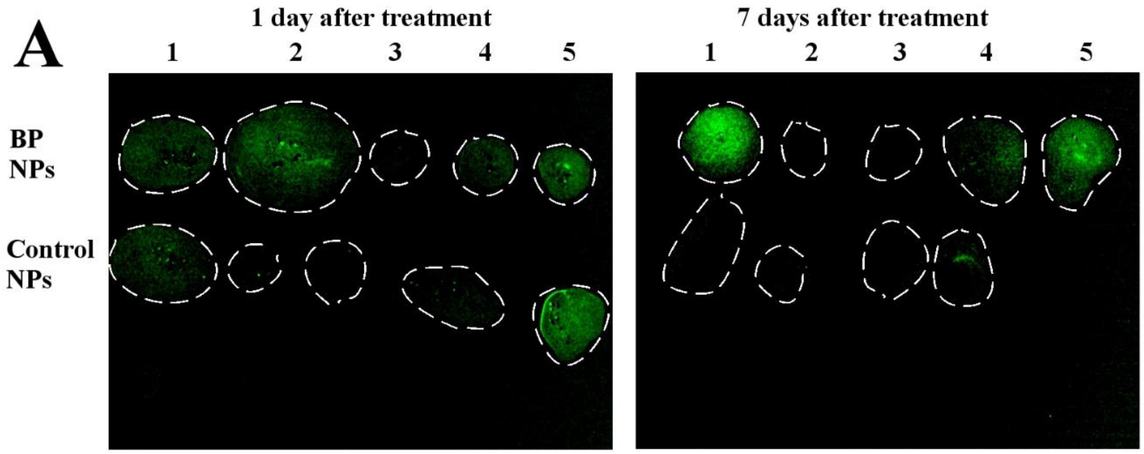


**B** ***Measurement method:***

**Convert picture to B&W image**

↓

**Select specific tumour**

↓

**Measure tumour fluorescence by 'Integrated density' function**

↓

**Continue to the next tumour**

**Figure S4. Bright field images of Saos-2 cells** following 4h incubation with A) doxorubicin-conjugated BP NPs (0.1 mg/ml), B) BP NPs (0.1 mg/ml) and C) untreated cells. The cells nuclei were stained with Hoechst (blue).

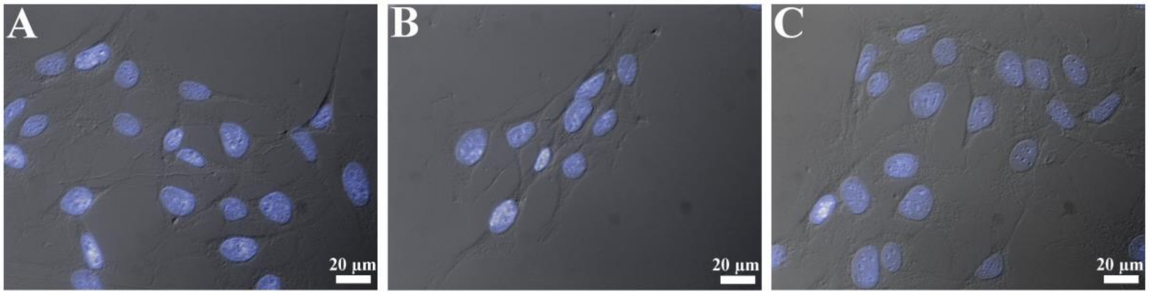

Supplement: Supplementary file 1 — Additional file 1. Additional figures. [file 12951_2016_233_MOESM1_ESM.pdf]
